# Myelonecrosis: A Clinicopathological Study from a Tertiary Care Center in South India over a Twelve-Year Period

**DOI:** 10.1155/2014/890510

**Published:** 2014-01-23

**Authors:** Jinkala Sree Rekha, Rakhee Kar, Debdatta Basu

**Affiliations:** Department of Pathology, Jawaharlal Institute of Postgraduate Medical Education and Research (JIPMER), Puducherry 605006, India

## Abstract

*Aims*. To study the etiology, diagnostic features, and clinical significance of myelonecrosis. *Methods*. A retrospective review of all trephine biopsies done over 12 years (January 2000 to December 2012) in Department of pathology was done and all trephine biopsies showing MN were identified and studied. *Results*. Twenty-five cases accounting for 0.4% were identified. Fever and generalized weakness were the common presenting symptoms. Anemia was seen in all cases followed by thrombocytopaenia. Malignancy was the underlying cause in 64% of cases; hematolymphoid malignancy was seen in two-thirds and solid malignancies in one-third of the cases. Tuberculosis accounted for 16% of the cases and the etiology was unknown in 12%. *Conclusions*. The causes of MN are varied and hematological malignancy and solid malignancies are the most common causes. Presence of myelonecrosis is associated with a poor prognosis. Myelonecrosis may obscure the underlying disorder and hence a thorough search in the bone marrow biopsy itself with the help of immunohistochemistry may prove worthwhile in identifying the underlying disease.

## 1. Introduction

Myelonecrosis (MN) or bone marrow necrosis is a rare and unique clinicopathological entity. It is characterized by the necrosis of the medullary stroma and hematopoietic cells in large areas of bone marrow [1–3]. It is seen in the trephine biopsy as amorphous eosinophilic areas with poorly defined necrotic cells, which may or may not be accompanied by necrosis of the cortical bone [4]. Though it is usually described in autopsy reports, it is uncommonly seen in antemortem trephine biopsies also. The causes of MN are multiple, hematolymphoid malignancy and metastasis by solid tumors being the most common underlying etiology.

In this study, the cases of MN were evaluated to study their etiology, diagnostic features, and clinical significance.

## 2. Materials and Methods

This is a descriptive study; a retrospective review of all trephine biopsies done over 12 years (January 2000 to December 2012) in Department of pathology was done and all trephine biopsies showing MN were identified. Both antemortem and postmortem trephine biopsies were included in the study. Postmortem biopsies were done in cases where peripheral smear findings suggested hematological abnormality; however patients expired before a bone marrow could be done, so postmortem trephine biopsy was done as a part of minimally invasive autopsy procedure followed in the institute.

Trephine biopsies showing extensive necrosis were included and cases showing granulomas with central caseation were excluded from the study. The bone marrow examination was done as a part of diagnostic work-up and written informed consent was taken from all the patients as per the institution protocols.

Bone marrow aspiration and biopsy were done from the posterior superior iliac spine. Peripheral smear, bone marrow aspiration slides were stained with Giemsa. The trephine biopsies were stained with haematoxylin and eosin. Special stains like Periodic Acid Schiff (PAS), Gomori-Methenamine Silver (GMS) for fungus, Ziehl-Neelsen Staining for Acid fast bacilli, and immunohistochemistry (IHC) was done in the trephine biopsies wherever necessary. The clinical, peripheral blood, and bone marrow findings of these cases were reviewed and analysed. The results are expressed as percentage of total number of cases.

## 3. Results

Between January 2000 to December 2012, MN was diagnosed in 25 samples among 6,010 trephine biopsies accounting for 0.4%. All the cases were above 12 yrs of age. Median age was 33 years (range 13–57 years) and five patients were females. In 23 cases, antemortem BM aspiration and biopsies were done. In two postmortem cases only trephine biopsy was available.

The most common presenting symptom was fever seen in 17 patients (68%) followed by generalized weakness in six patients (24%). Bone pain was seen in only one patient in our study. The most common sign was pallor seen in 10 patients (40%). Generalized lymphadenopathy was seen in 7 (28%) and SVC obstruction in two patients.

The peripheral blood findings are tabulated in Table 1. Anemia was present in all the patients and in 48% was severe (Hb less than 6 gm/dL). Bicytopenia (anemia with thrombocytopaenia) was seen in 13 (52%) of patients. Pancytopenia seen in 28% and leucoerythroblastic blood picture seen in 32% were the other common peripheral blood findings.

In 10 (40%) of our cases BM aspiration was a particulate and unsatisfactory for opinion. In six more cases, BM aspiration showed evidence of MN in the form of amorphous granular material with degenerated cells and further interpretation was not possible. The trephine biopsy showed extensive areas of MN in all the cases.

### 3.1. Underlying Diseases Causing MN

**Figure 1:**
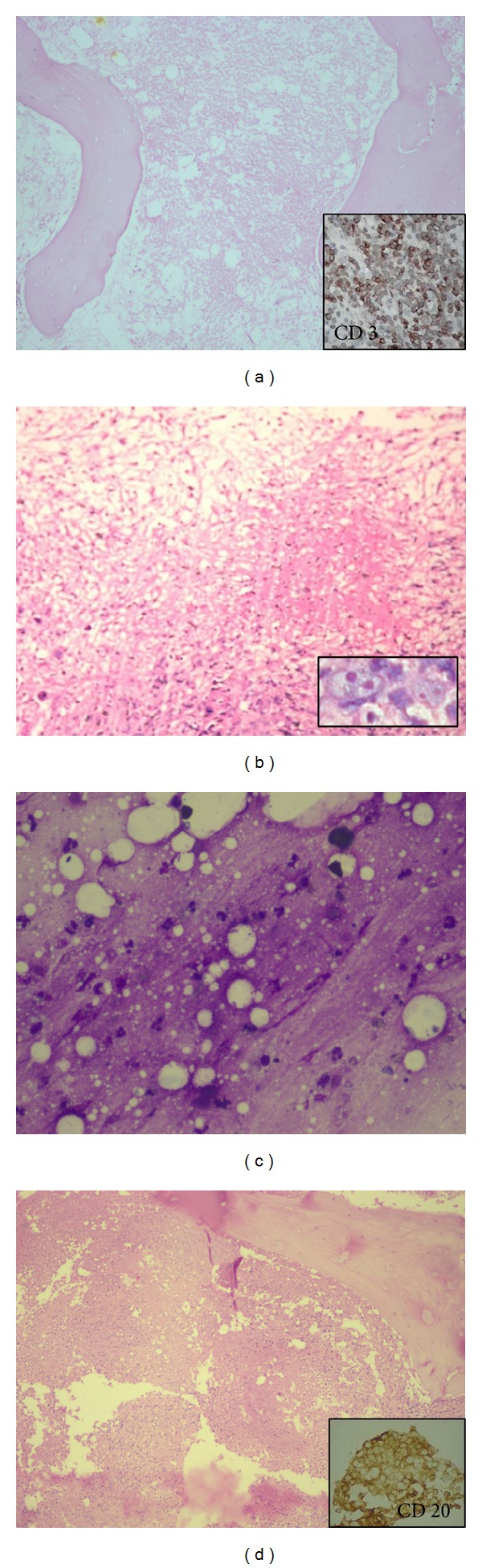
Trephine biopsies showing myelonecrosis: (a) acute lymphoblastic leukaemia (H&E 20X), inset showing CD 3 positivity (DAB 400X); (b) Hodgkin lymphoma (H&E 40X), inset showing Reed Sternberg cell (H&E 400X); (c) and (d) NHL-DLBCL, bone marrow aspirate showing amorphous granular material suggesstive of myelonecrosis ((c), Giemsa 400X), biopsy with myelonecrosis ((d), H&E 40X), and inset showing CD20 positivity (DAB 400X).

The distribution of various underlying diseases causing MN is shown in Table 2. The most common underlying disease causing MN was malignancy seen in 16 patients (64%). Hematolymphoid malignancy was seen in 11 patients (Figure 1(a)). We had four patients of ALL and two patients of AML. Among these, five patients showed evidence of MN at presentation. Only one patient of ALL showed extensive areas of MN after chemotherapy. Five of these cases showed leucocytosis with blasts in the peripheral blood. One case of AML, however, showed pancytopenia with presence of myeloblasts in the peripheral blood.

Four cases of lymphomas showed extensive areas of MN in the marrow (Figures 1(b), 1(c), and 1(d)). Four cases were of Diffuse large B cell lymphoma of which three showed MN after chemotherapy. In three of these cases bone marrow was involved by lymphoma which was proved by IHC using CD 20 and in one case infiltration by lymphoma was not seen. One of these patients was also HIV reactive. One case of Hodgkin lymphoma-lymphocyte depleted type showed MN at presentation.

**Figure 2:**
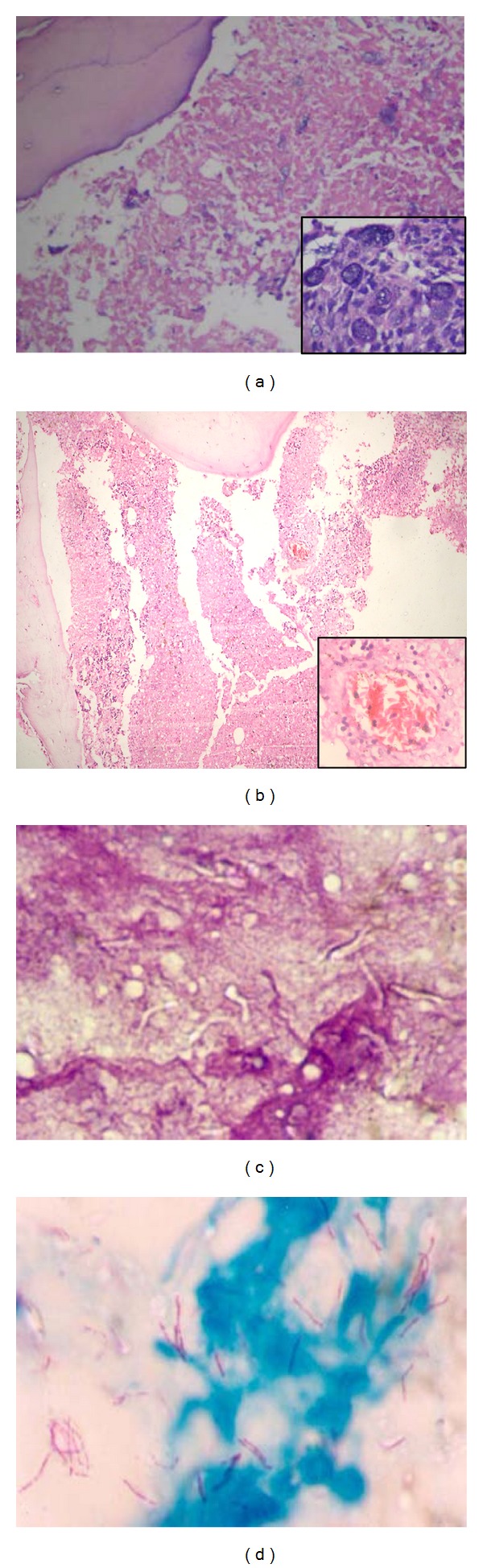
Trephine biopsies showing myelonecrosis: (a) metastatic adenocarcinoma (H&E 100X), inset showing epithelial cells with mucin secretion (H&E 400X); (b) sickle cell anemia (H&E 40X), inset showing blood vessel occluded with sickle cells (H&E 400X); (c) and (d) retropositive patient with imprint showing myelonecrosis with negative images ((c), H&E 400X) with strong positivity for acid fast bacilli in Ziehl-Neelsen staining ((d), Ziehl-Neelsen 400X).

Among the nonhaematological malignancies, metastatic adenocarcinoma with an unknown primary was seen in four patients. In all these cases metastatic adenocarcinoma was initially identified on the trephine biopsy (one was a postmortem biopsy) with extensive areas of MN (Figure 2(a)). We have in our series, a case of metastatic melanoma to the bone marrow showing areas of MN. Bone marrow was done in all cases due to the presence of leucoerythroblastic blood picture.

Among the nonhaematological causes, tuberculosis was the commonest cause seen in four patients (16%). MN was appreciated on the aspirate in two cases. Three of these patients were HIV reactive and all three cases were strongly positive for acid fast bacilli on ZN staining (Figures 2(c) and 2(d)). In one case, in addition to MN, haemophagocytosis was also seen. One HIV reactive patient presenting with pancytopenia showed MN only; no granulomas were identified.

A known case of sickle cell anemia showed extensive areas of MN in the postmortem trephine biopsy (Figure 2(b)). In three cases the cause of MN could not be identified on the biopsy. In two cases, the viable marrow was showing reactive nonspecific changes; no atypical cells or granulomas were identified. In one case however there were few atypical cells seen, which were positive for myeloperoxidase immunostain. The peripheral blood showed bicytopenia with 8% blasts, but a definitive diagnosis could not be reached as there was extensive MN.

An attempt was made to type the necrosis as coagulative, fibrinous, or caseous. However, in the majority of our cases coagulative necrosis was seen. In few cases, like tuberculosis and HIV reactive patients with tuberculosis, the exact typing of necrosis was not possible.

### 3.2. Outcome

Seven of these patients expired (one AML and three NHL), three of the cases of metastatic adenocarcinoma deferred any treatment, and two cases of ALL underwent chemotherapy are alive and doing well. The remaining cases are lost to followup.

## 4. Discussion

MN was first described in a patient with sickle cell disease by Wade and Stevenson in 1941 [5]. The relative frequency of MN is 0.37% to 6.5% [3–6]. The most common cause is leukaemias and lymphomas followed by solid malignancies involving the BM [7]. It has also been described after chemotherapy, due to the effect of certain drugs used mainly in haematooncology and also due to various infectious agents [7].

Basu and Yaranal had done a similar study in our institute, which included seven cases [8]. The present study adds upon the previous study and discusses the diseases causing MN over a twelve year period. In this study, we have included only trephine biopsies showing extensive necrosis, usually greater than 20% of the marrow. We would like to emphasize again that all cases of tuberculosis showing granuloma with central focus of caseation were excluded from this study.

The presenting features of MN are nonspecific. The most common symptom of myelonecrosis described in the literature is bone pain [7]. This was however seen in only one case in our study. Prajapati et al. and Norgard et al. also did not find bone pain as the presenting symptom in their patients [2, 9]. Bone pain that occurs in MN is described as an acute, intense debilitating pain that is usually located in the lowerback [7]. The second most common presenting symptom is fever, seen in our study in 68%. Fever as the common presenting feature was also described in other studies [8, 10]. Fever can be attributed to the pyrogens released from the necrotic marrow.

The most common peripheral smear findings described in MN are anemia and thrombocytopenia as seen in our study, in 100% and 76% of cases [7]. These may or may not be associated with a leucoerythroblastic blood picture. In our study, 5/6 cases of leukaemias and 5/5 cases of metastatic malignancies presented with anemia and thrombocytopaenia. All these five cases of metastasis showed an associated leucoerythroblastic blood picture. Pancytopenia was seen 29.2% of our cases and is also a significant peripheral blood finding. Haematological abnormalities necessitate bone marrow examination and often give a clue to diagnosis [3, 6].

Our study is in correlation with other studies in the literature by Paydas et al. and Elgamal et al. that hematolymphoid malignancies account for two-thirds of the cases of MN and solid malignancies account for one-third [10, 11]. Among the hematolymphoid malignancies, acute lymphoblastic leukaemia (ALL) is the most common. ALL can present with MN at presentation, after induction or at relapse. AML presents with MN usually at presentation. High grade lymphomas like DLBCL as well as indolent lymphomas like chronic lymphocytic leukemia or hairy cell leukemia can also present with MN, usually after chemotherapy. MN is less commonly associated with chronic myeloproliferative neoplasms [7].

The association of MN with solid tumors is also known. Often, the primary origin of the tumor is not found, even after an extensive search [7].

MN can even precede leukaemias. Niebrugge and Benjamin describe two patients who initially presented with MN and subsequently developed ALL emphasizing the need for close monitoring of patients with unexplained myelonecrosis [12]. In patients with leukaemia and metastatic carcinoma, development of severe pancytopenia with or without chemotherapy and which does not respond to supportive measures, the possibility of myelonecrosis should be considered [3, 6].

Most common infectious cause of MN in our study was tuberculosis (TB). Tuberculosis shows varied clinical as well as haematological manifestations. Anemia is the most common manifestation but other abnormalities like leucopenia, thrombocytopenia, monocytosis, basophilia, disseminated intravascular coagulopathy, leukaemoid reaction, thrombocytosis, or a pancytopenia can also occur [13]. Pancytopenia is associated with a poor prognosis in TB [14]. Mangion and Schiller state that leukaemoid reaction and pancytopenia with TB may represent haematological disease with opportunistic mycobacterial infection as was seen in one of our case [15].

There are many other causes of MN. Paydas et al. has reported MN in antiphospholipid antibody syndrome [16]. Laso et al. has reported MN in association with tumor emboli and disseminated intravascular coagulation [17]. Septic emboli by infective endocarditis, sepsis by Gram positive and Gram negative bacteria, hemolytic uremic syndrome, and hyperparathyroidism are among the rare causes of MN [7].

The pathogenesis of myelonecrosis is controversial and subject to debate [3]. It could be due to infiltration by neoplastic cells and decreased oxygen tension due to increased proliferative activity of tumour cells, elaboration of tumour necrosis factor, vasoocclusion, and thrombosis, radiation injury or the effect of chemotherapy [3, 6, 8, 18, 19]. It can be seen prior to chemotherapy as seen in five of our cases of leukaemia and may be due to occlusion of medullary nutrient vessels by blasts [2, 6, 8]. In disseminated tuberculosis the toxic effects on the marrow by large amounts of tuberculin protein may lead to marrow necrosis [8].

The presence of myelonecrosis is a bad prognostic sign. Cassileth and Brooks reported that MN after induction chemotherapy indicates a poor prognosis [1]. There are reports in the literature of marrow regeneration after extensive MN especially in cases of ALL [20]. We also have a case of ALL who recovered inspite of extensive MN at presentation and is in remission and doing well now. Janssens et al. suggests that BMN is not always an ominous sign and that vigorous supportive care together with disease specific treatment must be continued to permit adequate time for spontaneous recovery of the normal hematopoietic tissue [7].

As the underlying causes of MN are varied, the disease process may get obscured by extensive necrosis of the marrow. In our study 40% of cases showed diluted marrow aspirates and MN was seen in only six cases on the aspirate smears. The final diagnosis was made only on the trephine biopsy in all the cases, after having to resort to deeper cuts, histochemistry, and application of IHC. Trephine biopsy provides the pathologist with material for taking deeper sections to identify a viable focus of cells, to do IHC to identify the possible primary in malignancies and immunophenotyping in hematolymphoid malignancies. Some immunohistochemical markers like CD45, CD3, CD20, S100, and cytokeratin AE1AE3 retain reactivity even in the necrosed ghost cells, thus helping the pathologist in identifying the underlying disease.

The most practical approach when encountered with such cases would be to get a good and satisfactory knowledge of clinical history, laboratory and instrumental data and a painstaking histological evaluation.The histological examination includes taking deeper sections and performing immunohistochemistry by using those antibodies that retain reactivity on ghost dead cells. All these together serve as the the most useful tools that permit to identify and classify that “invisible pathology,” which is not so infrequently encountered.

Our study confirms that causes of MN are varied and haematopoietic malignancy is the most common cause. The clinical features of MN are nonspecific. Haematological abnormalities are commonly present and are clue to the diagnosis. Myelonecrosis may obscure the underlying disorder and hence a thorough search in the bone marrow biopsy itself may be the key to diagnosis.

## Figures and Tables

**Table 1:** Peripheral blood findings in patients with myelonecrosis.

Peripheral smear findings	*n* = 25 (%)
Anemia	25 (100%)
Severe anemia (Hb < 6 gm%)	12 (48%)
Bicytopenia	13 (52%)
Anemia + thrombocytopaenia	12 (48%)
Pancytopenia	7 (28%)
Anemia only	4 (16%)
Leucoerythroblastic picture	8 (32%)
Microangiopathic hemolytic anemia	1 (4%)
Leukemia	5 (20%)

**Table 2:** Distribution of underlying disease in patients with myelonecrosis.

Distribution of underlying disease	No. of cases (*n* = 25)
Malignant (16 cases; 64%)	
Leukemia	6 ( ALL-4; AML-2)
Lymphoma	5 (NHL-4; HL-1)
Metastasis	5 (adeno ca.-4, melanoma-1)
Nonmalignant (6 cases; 24%)	
TB with HIV	3
TB with hemophagocytosis	1
HIV	1
Sickle cell anemia	1
Unknown cause (3 cases; 12%)	
—	3
